# [Retracted] Increased resistin suggests poor prognosis and promotes development of lung adenocarcinoma

**DOI:** 10.3892/or.2023.8595

**Published:** 2023-06-29

**Authors:** Cui-Cui Zhao, Jing Chen, Rui-Fang Niu, Yan Liu, Chuan-Gui Zhang

Oncol Rep 40: 3392–3404, 2018; DOI: 10.3892/or.2018.6736

Following the publication of this paper, it was drawn to the Editor's attention by a concerned reader that the colony formation assay data shown in Fig. 3A on p. 3399 were strikingly similar to data that were already under consideration for publication in another article written by different authors at different research institutes. Owing to the fact that the contentious data in the above article were already under consideration for publication prior to its submission to *Oncology Reports*, the Editor has decided that this paper should be retracted from the Journal. The authors were asked for an explanation to account for these concerns, but the Editorial Office did not receive a satisfactory reply. The Editor apologizes to the readership for any inconvenience caused.

